# Machine-learning-assisted target prioritization and experimental validation of the neoeriocitrin–mTOR axis in impaired osteogenic differentiation

**DOI:** 10.3389/fphar.2026.1955945

**Published:** 2026-10-30

**Authors:** Yuhao Wang, Jianguang Luo

**Affiliations:** Spinal Surgery Department, Ningbo Zhenhai People’s Hospital (Ningbo Seventh Hospital), Ningbo City, Zhenhai District, China

**Keywords:** autophagy, machine learning, mTOR, neoeriocitrin, osteogenic differentiation, osteoporosis, target validation

## Abstract

Neoeriocitrin (NEO) is a citrus-derived flavonoid with potential osteoprotective activity, but its pharmacological targets and mechanisms in impaired osteogenic differentiation remain unclear. Osteoporosis-related differentially expressed genes were intersected with predicted NEO targets, and least absolute shrinkage and selection operator regression, random forest, and support vector machine analyses consistently prioritized MTOR for mechanistic investigation. Molecular docking predicted a favorable binding pose of NEO at the 12-kDa FK506-binding protein (FKBP12)–mTOR FKBP12–rapamycin-binding (FRB) interface, while the cellular thermal shift assay (CETSA) demonstrated increased cellular thermal stability of mTOR following NEO treatment, supporting an mTOR-associated intracellular effect. In dexamethasone-treated bone marrow mesenchymal stem cells, NEO dose-dependently restored alkaline phosphatase activity, mineralized nodule formation, and the expression of runt-related transcription factor 2 (RUNX2), osteopontin (OPN), and alkaline phosphatase (ALP). NEO also enhanced autophagic flux, increased the microtubule-associated protein 1 light chain 3 beta (LC3B)-II/LC3B-I ratio, reduced p62 expression, and suppressed dexamethasone-induced phosphorylation of mechanistic target of rapamycin (mTOR) and ribosomal protein S6 kinase 1 (S6K1). Pharmacological inhibition of autophagy with 3-methyladenine (3-MA) and mTOR overexpression both attenuated the pro-osteogenic effects of NEO. Collectively, these findings support an mTORC1-associated S6K1 signaling mechanism linking NEO treatment to autophagy regulation and osteogenic differentiation. NEO may therefore represent a candidate natural compound for further preclinical investigation in osteoporosis-related bone loss.

## 1 Introduction

Osteoporosis (OP) is a systemic skeletal disorder characterized by reduced bone mass and deterioration of bone microarchitecture, resulting in increased bone fragility and fracture susceptibility (Compston et al., 2019). It is a major global public health concern (Kanis et al., 2019; Clynes et al., 2020). Fragility fractures are associated with persistent pain, impaired physical function, reduced quality of life, and increased mortality (Compston et al., 2019; Clynes et al., 2020). OP also imposes a substantial economic burden on healthcare systems and society (Hernlund et al., 2013). Current antiresorptive treatments include bisphosphonates and inhibitors of receptor activator of nuclear factor kappa B ligand (RANKL) (Eastell et al., 2019). Long-term safety concerns, treatment adherence, and medication costs remain challenges (Eastell et al., 2019; Tsourdi et al., 2017). These limitations support the search for additional therapeutic approaches (Eastell et al., 2019; Tsourdi et al., 2017; Yang et al., 2025). Recent experimental work has also examined environmental contributions to skeletal injury, including altered bone physicochemical properties after subchronic herbicide exposure in female rats (Hamdaoui et al., 2025). Bone homeostasis depends on a balance between osteoblast-mediated bone formation and osteoclast-mediated bone resorption (Florencio-Silva et al., 2015). Experimental studies of skeletal metastases likewise illustrate the relationship between bone microarchitecture and the local biological environment (Badraoui et al., 2022). Bone marrow mesenchymal stem cells (BMSCs), the principal precursors of osteoblasts, contribute to this balance through their capacity for osteogenic differentiation (Florencio-Silva et al., 2015; Zhang L. et al., 2025). Strategies that preserve or enhance osteoblast formation therefore warrant investigation in OP.

Natural compounds have attracted interest as potential candidates for OP research (Cai et al., 2026), although their pharmacological mechanisms and safety require careful evaluation. Neoeriocitrin (NEO) is a citrus-derived flavonoid with reported osteogenic activity (Li et al., 2011). Previous studies have examined its effects on osteoblast-lineage cells and human dental pulp stem cells (Li et al., 2011; Wu et al., 2025). NEO has been reported to increase osteogenic markers, including runt-related transcription factor 2 (RUNX2), type I collagen, and osteocalcin (OCN), in MC3T3-E1 cells (Li et al., 2011). Flavonoids from Rhizoma Drynariae have also been associated with changes in sclerostin (SOST) and RANKL in osteocyte models (Jin et al., 2022). However, findings for mixed flavonoid preparations should not be attributed specifically to NEO. The effects of NEO on BMSC osteogenic differentiation and its efficacy and safety in osteoporosis models *in vivo* remain to be established.

We used database mining and complementary machine-learning algorithms to prioritize candidate mechanisms underlying the effects of NEO. Autophagy, a conserved process that degrades and recycles intracellular components, contributes to stem cell homeostasis and skeletal physiology (Mizushima and Komatsu, 2011; Galluzzi and Green, 2019). In BMSCs, autophagy influences cellular remodeling, oxidative stress, and differentiation-related signaling (Song et al., 2022). Appropriate autophagic activity supports osteogenic differentiation, whereas impaired autophagy can compromise BMSC function and bone homeostasis (Qi et al., 2017). Mechanistic target of rapamycin (mTOR) forms two distinct signaling complexes, mTOR complex 1 (mTORC1) and mTOR complex 2 (mTORC2) (Saxton and Sabatini, 2017). mTORC1 regulates autophagy initiation through substrates such as unc-51-like autophagy activating kinase 1 (ULK1); phosphorylation of ribosomal protein S6 kinase 1 (S6K1) at Thr389 is a downstream readout of mTORC1 signaling (Saxton and Sabatini, 2017; Kim et al., 2011). mTORC2 has distinct functions, including regulation of AKT phosphorylation at Ser473 (Saxton and Sabatini, 2017). The effects of mTOR signaling on skeletal cells depend on cell type and biological context (Chen and Long, 2018). In some experimental settings, excessive mTOR-associated signaling is linked to impaired autophagy and BMSC osteogenesis (Lin et al., 2021). The present study focuses on the mTORC1-associated S6K1 signaling arm and autophagy, while recognizing that the available experiments do not establish complex-selective effects of NEO.

Building on this background, the present study established a dexamethasone (DEX)-induced model of impaired osteogenic differentiation in BMSCs to systematically evaluate the effects of NEO on early osteogenic activity and late-stage mineralization. Integrated bioinformatics analyses based on public databases were employed to predict and identify key molecular targets of NEO in OP. Furthermore, a combination of computational analyses and *in vitro* experiments was carried out. These experiments were used to elucidate the interplay between NEO, the mTOR signaling pathway, its phosphorylation status, and protective autophagy, thereby clarifying the molecular basis by which NEO alleviates impaired osteogenic differentiation in BMSCs. This study provides a machine-learning-assisted and experimentally validated pharmacological framework for investigating the osteoprotective effects of a natural small-molecule compound and supports further preclinical evaluation of NEO; it does not establish efficacy in animals or patients.

## 2 Materials and methods

### 2.1 Reagents and drugs

Neoeriocitrin (NEO, purity ≥98%, CAS No. 13241–32–2, PubChem CID: 114627, Yuanye Bio-Technology Co., Ltd., Shanghai, China); Dexamethasone (DEX, Sigma-Aldrich, St. Louis, MO, USA); 3-Methyladenine (3-MA, Sigma-Aldrich, St. Louis, MO, USA); Fetal Bovine Serum (FBS, Gibco, Thermo Fisher Scientific, Waltham, MA, USA); Penicillin-Streptomycin (Gibco, Thermo Fisher Scientific, Waltham, MA, USA); Ascorbic acid (Sigma-Aldrich, St. Louis, MO, USA); β-Glycerophosphate disodium salt hydrate (Sigma-Aldrich, St. Louis, MO, USA).

ALP Staining Kit (Solarbio Science & Technology Co., Ltd., Beijing, China); Alizarin Red S (ARS, Solarbio Science & Technology Co., Ltd., Beijing, China); Bicinchoninic acid (BCA) Protein Quantification Kit (Beyotime Biotechnology, Shanghai, China); mCherry-EGFP-LC3 Adenovirus (GenePharma Co., Ltd., Shanghai, China).

RUNX2 (Rabbit, 1:1000, 20700-1-AP); OPN (Rabbit, 1:1000, 22952-1-AP); ALP (Rabbit, 1:1000, 11187-1-AP); LC3B (Rabbit, 1:1000, 14600-1-AP); p62 (Rabbit, 1:5000, 18420-1-AP); mTOR (Mouse, 1:10000, 66888-1-Ig); p-mTOR (Ser2448) (Rabbit, 1:2000, 80596-1-RR); S6K1 (Rabbit, 1:1000, 14485-1-AP); p-S6K1 [Phospho-p70 (S6K) (Thr389)/p85 (S6K) (Thr412)](Rabbit, 1:1000, 28735-1-AP); GAPDH (Mouse, 1:10000, 60004-1-Ig); HRP-conjugated Affinipure Goat Anti-Rabbit IgG (H + L) (Goat, 1:5000, SA00001-2); HRP-conjugated Affinipure Goat Anti-Mouse IgG (H + L) (Goat, 1:5000, SA00001-1) were manufactured by Proteintech Group (Rosemont, IL, USA).

### 2.2 Bioinformatics analysis

#### 2.2.1 Data acquisition

The transcriptomic dataset associated with OP was obtained from the National Center for Biotechnology Information (NCBI) Gene Expression Omnibus (GEO) database (GSE7429). The dataset comprises circulating B lymphocytes from 20 postmenopausal women, including 10 individuals with high bone mineral density (BMD) as the control group (CON) and 10 individuals with low BMD as the OP group. The simplified molecular-input line-entry system (SMILES) representation of NEO (PubChem CID: 114627) was retrieved from the PubChem database and used for subsequent target prediction and molecular docking analyses.

#### 2.2.2 Differential gene expression screening

The GSE7429 dataset was normalized and corrected for batch effects prior to analysis. Differentially expressed genes (DEGs) associated with OP were then identified using the limma package in R, with selection criteria set at |logFC| > 1 and P < 0.05.

#### 2.2.3 Drug target prediction

NEO-related potential targets were predicted using the PharmMapper (https://www.lilab-ecust.cn/pharmmapper/index.html), SwissTargetPrediction (https://swisstargetprediction.ch/), SuperPred (prediction.charite.de) databases. After integrating the outputs from these platforms, duplicate entries were removed to obtain a non-redundant predicted target set for NEO.

#### 2.2.4 Core gene screening and functional enrichment analysis

The DEGs associated with OP were intersected with the predicted target set of NEO to identify core genes potentially involved in NEO-mediated intervention in OP. Subsequently, Gene Ontology (GO) functional enrichment analysis was performed for these core genes. The clusterProfiler package in R was used for this analysis. The goal was to explore their primary biological functions and associated signaling pathways.

#### 2.2.5 PPI network construction and key node screening

Core genes involved in NEO-mediated OP intervention were analyzed using the STRING database (https://string-db.org/). A protein–protein interaction (PPI) network was constructed based on *Homo sapiens* data. The confidence score was set to high (interaction score ≥0.7), and isolated nodes were removed from the network. The interaction data were exported in tab-separated values (TSV) format, and the network was visualized using Cytoscape version 3.9.1. Hub genes in the network were determined through the built-in CytoHubba plugin of Cytoscape, and these genes served as the core target candidates for further validation.

#### 2.2.6 Machine-learning-based candidate target prioritization

To prioritize candidate targets among the 19 genes obtained from the intersection of OP-associated differentially expressed genes and predicted NEO targets, three complementary machine-learning methods were applied: least absolute shrinkage and selection operator (LASSO) regression, random forest (RF), and support vector machine (SVM). LASSO regression was implemented using the glmnet package, and 10-fold cross-validation was used to select the penalty parameter λ. RF and SVM analyses were performed using the randomForest and e1071 packages, respectively. Candidate genes were ranked according to the feature-importance results generated by each method, and genes meeting the prespecified selection criterion were retained. Genes consistently prioritized by all three approaches were considered high-confidence candidates for subsequent mechanistic and experimental evaluation. These analyses were used for candidate-target prioritization rather than for the development or validation of a diagnostic classification model. Given that the analyses were restricted to a predefined set of 19 candidate genes, these machine-learning approaches were used as complementary prioritization tools rather than as unbiased target-discovery methods.

#### 2.2.7 Molecular docking

The structural data used for molecular docking were obtained from the RCSB Protein Data Bank (PDB ID: 3FAP; resolution: 1.85 Å). The 3FAP structure contains human 12-kDa FK506-binding protein (FKBP12; chain A, residues 1–107) and the FKBP12-rapamycin-binding (FRB) domain of human mTOR/FRAP (chain B, PDB residues 108–201), corresponding to residues 2019–2112 of the canonical human mTOR sequence. Both protein chains were retained as the receptor complex because the docking region encompassed the FKBP12–mTOR FRB interface. The structure was preprocessed using PyMOL by removing water molecules and the co-crystallized ligands. AutoDockTools was then used to prepare the receptor complex by adding hydrogens, assigning charges, and defining the grid box. Molecular docking of NEO to the FKBP12–mTOR FRB receptor complex was performed using AutoDock v4.2.6 with the semi-flexible docking strategy and the Lamarckian genetic algorithm (LGA). The predicted binding energy was used to rank docking poses rather than to determine an experimental binding affinity.

The docking search space was defined using a grid box centered at x = −11.223 Å, y = 24.366 Å, and z = 32.180 Å, with dimensions of 40 × 40 × 46 Å along the x-, y-, and z-axes, respectively. The pose with the lowest predicted binding energy was selected for visualization and interaction analysis. Residue assignments were verified according to the corresponding protein chains, and residues belonging to the mTOR FRB domain were mapped to the canonical human mTOR sequence.

### 2.3 Cell experiments

#### 2.3.1 Cell culture and grouping treatment

Primary rat bone marrow mesenchymal stem cells (BMSCs; catalog No. SCSP-402) were obtained from the Cell Bank of the Chinese Academy of Sciences. They were grown in Dulbecco’s modified Eagle medium/nutrient mixture F-12 (DMEM/F12) supplemented with 10% FBS and 1% penicillin-streptomycin under normal conditions (37 °C, 5% CO_2_). Fresh medium was changed every 2–3 days in order to keep an optimal environment for cell proliferation, and cells between passages 3 and 5 were used for further experiments. For osteogenic differentiation, BMSCs were cultured in osteogenic induction medium consisting of DMEM/F12 supplemented with 10% FBS, 1% penicillin–streptomycin, 50 μg/mL ascorbic acid, and 10 mM β-glycerophosphate disodium salt hydrate. The same osteogenic induction medium was used for all experimental groups.

To establish the DEX-induced model of impaired osteogenic differentiation, BMSCs were assigned to six groups: Control (Con), DEX, DEX + NEO-L, DEX + NEO-M, DEX + NEO-H, and NEO-H alone (NEO-H). The Control group received osteogenic induction without DEX or NEO. The DEX group was treated with 100 nM DEX. The DEX + NEO-L, DEX + NEO-M, and DEX + NEO-H groups were treated with 100 nM DEX together with 2.5, 5, and 10 μM NEO, respectively. The NEO-H-alone group received 10 μM NEO without DEX to evaluate the effect of NEO on basal osteogenic differentiation under osteogenic induction conditions. The control and DEX groups received an equivalent volume of dimethyl sulfoxide (DMSO) as a vehicle control. Treatment-containing medium was refreshed every 3 days. The concentration of DEX was selected based on previous studies demonstrating that glucocorticoid exposure at nanomolar concentrations induces impaired osteogenic differentiation and alters the fate of BMSCs (Weinstein, 2012; Li et al., 2013).

For the functional rescue experiments, BMSCs were transfected with an mTOR overexpression construct (oe-mTOR) or the corresponding empty-vector control (oe-NC) and divided into four groups: oe-NC, oe-NC + NEO, oe-mTOR, and oe-mTOR + NEO. All groups were exposed to 100 nM DEX, while the NEO-treated groups received an additional 10 μM NEO. At 6 h after transfection, the medium was replaced with fresh complete medium, followed by an additional 24 h incubation before the indicated treatments were initiated.

#### 2.3.2 ALP staining (alkaline phosphatase staining)

Cells were seeded into 6-well plates and subjected to the designated treatments for 7 days. The medium was removed, and the cells were washed twice with phosphate-buffered saline (PBS) and fixed with 4% paraformaldehyde for 30 min. After further washing, ALP staining was performed according to the kit instructions. Images were acquired using an inverted microscope, and the ALP-positive area was quantified using ImageJ.

#### 2.3.3 Alizarin Red S staining

After 21 days of treatment, cells were washed with PBS, fixed with 4% paraformaldehyde for 30 min, and washed again. Cells were incubated with 0.1% ARS at room temperature for 30 min, and excess dye was removed by washing with PBS. Mineralized nodules were imaged using an inverted microscope, and the ARS-positive area was quantified using ImageJ.

#### 2.3.4 Cell viability assay

Cell viability was evaluated using a Cell Counting Kit-8 (CCK-8) assay. Briefly, BMSCs were seeded into 96-well plates and subjected to the indicated treatments. At 24 h and 48 h after treatment, CCK-8 reagent was added and incubated according to the manufacturer’s instructions. Absorbance was measured at 450 nm using a microplate reader. Cell viability was calculated relative to the control group.

#### 2.3.5 Western blot assay

After treatment, cells were washed twice with PBS and lysed on ice for 30 min in radioimmunoprecipitation assay (RIPA) buffer containing protease and phosphatase inhibitors. Lysates were centrifuged at 12,000 rpm for 15 min, and supernatants were collected. Protein concentrations were measured using a BCA kit. Equal amounts of protein were separated by sodium dodecyl sulfate–polyacrylamide gel electrophoresis (SDS-PAGE) and transferred to polyvinylidene fluoride (PVDF) membranes. Membranes were blocked with 5% nonfat milk for 1 h at room temperature and incubated with primary antibodies overnight at 4 °C. After three washes with Tris-buffered saline containing Tween 20 (TBST), membranes were incubated with the appropriate secondary antibodies for 1 h. Bands were detected by enhanced chemiluminescence (ECL), and band intensities were quantified using ImageJ. The pathway readouts were mTOR phosphorylation at Ser2448 and S6K1 phosphorylation at Thr389, assessed relative to their respective total protein levels. S6K1 Thr389 phosphorylation was interpreted as an mTORC1-associated downstream readout; mTOR Ser2448 phosphorylation alone was not considered sufficient to distinguish mTORC1 from mTORC2. No mTORC2-specific activity assay was performed.

#### 2.3.6 Autophagic flux detection

BMSCs were infected for 12 h with an adenovirus expressing mCherry–enhanced green fluorescent protein (EGFP)–LC3, subjected to the indicated treatments, and fixed with 4% paraformaldehyde. Images were acquired using a laser confocal microscope. Yellow puncta (mCherry+/EGFP+) represented autophagosomes, whereas red-only puncta (mCherry+/EGFP−) represented autolysosomes. Red-only puncta were counted using ImageJ as a readout of autophagic flux.

#### 2.3.7 Cellular thermal shift assay (CETSA)

BMSCs were treated with 10 μM NEO or an equivalent volume of DMSO for 1 h. Approximately 1 × 107 cells per treatment condition were collected and equally divided into eight aliquots, which were heated at 32, 37, 42, 47, 52, 57, 62, or 67 °C for 10 min and immediately cooled on ice. Cells were lysed by three freeze/thaw cycles, after which the samples were centrifuged at 20,000 × g for 20 min at 4 °C to collect soluble protein-containing supernatants. Western blot analysis was performed to evaluate soluble mTOR protein levels at different temperatures. For each independent experiment, the mTOR signal at each temperature was normalized to the mTOR signal at 32 °C within the same treatment group, which was defined as 100%. GAPDH was displayed as a reference protein but was not used as a temperature-invariant normalization control for CETSA quantification. Three independent experiments were performed. Thermal stability was quantitatively evaluated by calculating the area under the thermal stability curve (AUC) over 32 °C–67 °C using the trapezoidal method in GraphPad Prism 8.0. Paired AUC values obtained from DMSO- and NEO-treated samples within the same independent experiment were compared using a two-tailed paired Student’s t-test.

### 2.4 Statistical analysis

Unless otherwise stated, n = 3 represents three independently performed cell-culture experiments, each initiated, cultured, treated, and analyzed separately. Technical replicate wells, microscopic fields, or repeated measurements within the same experiment were averaged to generate one value for each independent experiment. All experimental data were expressed as mean ± standard deviation (SD). Statistical analyses were performed using GraphPad Prism 8.0 software. For CETSA, AUC values obtained from paired DMSO- and NEO-treated samples within the same biological experiment were compared using a two-tailed paired Student’s t-test. For comparisons involving three or more experimental groups, one-way analysis of variance (ANOVA) followed by the appropriate *post hoc* test was used. A P value <0.05 was considered statistically significant.

## 3 Results

### 3.1 NEO alleviates DEX-induced inhibition of osteogenic differentiation in BMSCs

The development of OP is closely associated with impaired osteogenic differentiation of BMSCs, which represents a central event driving bone loss. Therefore, a DEX-induced model of osteogenic differentiation impairment in BMSCs was established to evaluate the protective effects of NEO on osteogenic differentiation potential (Li et al., 2013; Zhang K. et al., 2025). BMSCs were categorized into a CON group, a DEX-induced model group, and three NEO-treated experimental groups receiving 2.5, 5, and 10 μM, respectively, to evaluate dose-dependent effects. Cells were exposed to 100 nM DEX to induce osteogenic differentiation inhibition while simultaneously receiving the corresponding NEO treatments.

Before evaluating the osteogenic differentiation capacity, the effect of NEO on BMSC viability was assessed using the CCK-8 assay. Compared with the Control group, DEX exposure resulted in a mild reduction in cell viability, whereas NEO treatment at the indicated concentrations did not exhibit obvious cytotoxic effects. In addition, NEO-H alone showed comparable cell viability to the Control group, indicating that NEO did not adversely affect BMSC survival under the experimental conditions (Figures 1A,B).

**FIGURE 1:**
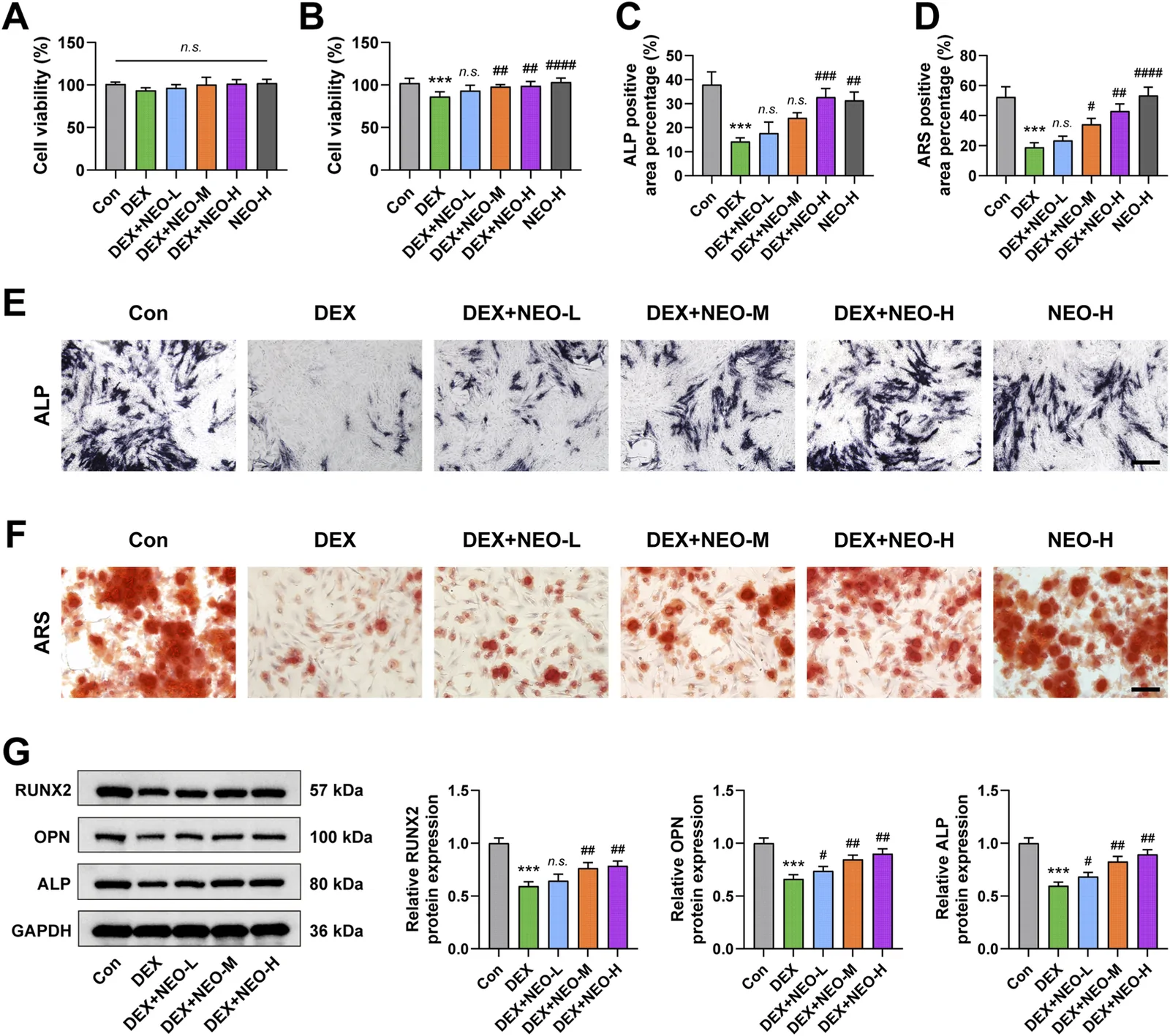
Neoeriocitrin alleviates DEX-induced inhibition of osteogenic differentiation in BMSCs. **(A,B)** Cell viability analysis of BMSCs treated with the indicated conditions for 24 h and 48 h using the CCK-8 assay. **(C,D)** Quantitative analysis of the ALP and ARS-positive area percentage. **(E)** Representative images of ALP staining after osteogenic induction. Scale bar: 200 μm. **(F)** Representative images of ARS staining for mineralized nodule formation in BMSCs. Scale bar: 100 μm. **(G)** Western blot analysis of osteogenic markers RUNX2, OPN, and ALP after the indicated treatments, with corresponding densitometric quantification. Data are presented as mean ± SD (n = 3 independent experiments). Statistical analysis was performed using one-way ANOVA followed by appropriate *post hoc* tests. ***P < 0.001 vs. the Con group; #P < 0.05, ##P < 0.01, ###P < 0.001, ####P < 0.0001 vs. the DEX group; n.s., not significant vs. the DEX group.

First, ALP staining was carried out after 7 days of DEX induction and drug treatment. This assay was employed to assess the early osteogenic differentiation capacity of BMSCs. DEX treatment significantly reduced the ALP-positive staining area in BMSCs. Following treatment with different concentrations of NEO, the ALP-positive area increased in a dose-dependent manner. Recovery was more evident in the medium- and high-dose groups (NEO-M and NEO-H). Compared with the model group, these groups showed statistically significant differences (Figures 1C,E). Notably, the NEO-H alone group showed an ALP staining pattern comparable to the Control group, suggesting that NEO itself did not impair basal osteogenic differentiation.

To assess the late-stage mineralization ability of BMSCs, ARS staining was carried out after 21 days of treatment to evaluate calcium nodule formation. The results indicated that DEX markedly impaired mineralization, leading to a significant reduction in ARS-positive staining area. In contrast, NEO treatment promoted the recovery of calcium nodule formation. This effect became more evident with increasing NEO concentrations. The strongest improvement was observed in the high-dose group (NEO-H) (Figures 1D,F). Similarly, NEO-H alone maintained mineralization capacity comparable to the Control group.

Western blot analysis showed that DEX reduced the levels of RUNX2, OPN, and ALP in BMSCs. NEO treatment partially restored their expression, with a more pronounced response at higher concentrations (Figure 1G). Together with the staining results, these findings indicate that NEO attenuates DEX-induced impairment of BMSC osteogenic differentiation. The viability results did not indicate overt cytotoxicity at the concentrations and treatment durations examined.

### 3.2 Bioinformatics analyses predicted that NEO is associated with the mTOR-related autophagy pathway in OP

Bioinformatics analyses were performed to identify candidate regulatory networks associated with OP and predicted NEO targets.

The GSE7429 dataset comprises circulating B lymphocytes from postmenopausal women with high or low BMD, rather than BMSCs. Differential expression analysis identified genes with higher and lower expression in the low-BMD group (Figure 2A), and the heatmap provides an overview of their expression patterns (Figure 2B). For identifiable candidate-gene profiles, Figure 3G presents labeled expression distributions for MTOR, AKT1, CCNE1, CES2, and ESR1. Because the source row identifiers and clustering order for Figure 2B were not available for reconstruction, individual gene identities cannot be assigned reliably to its heatmap rows. These cross-sectional expression differences support candidate prioritization but do not establish a causal role in OP or demonstrate the same expression changes in BMSCs.

**FIGURE 2:**
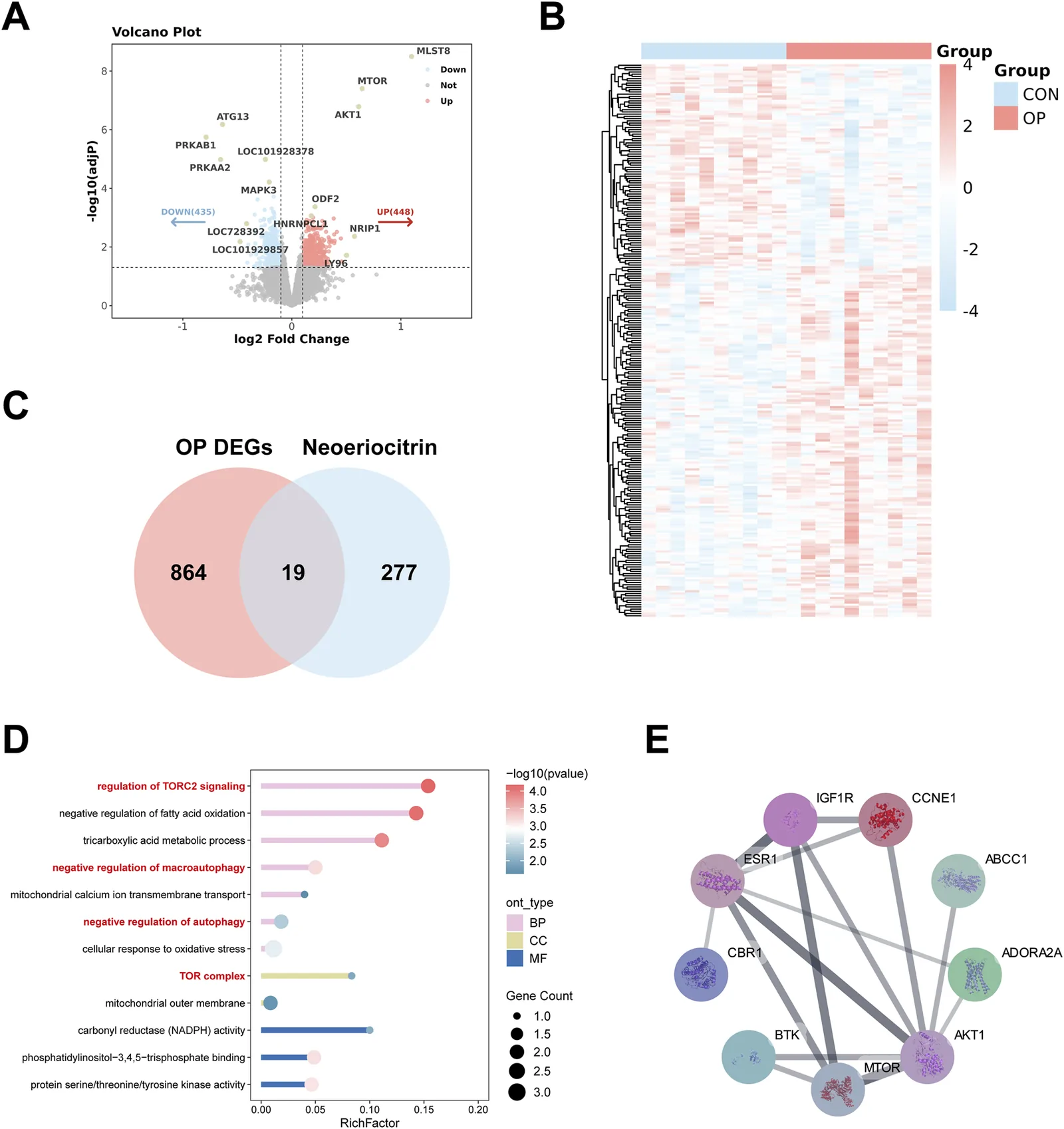
Bioinformatics prioritization of NEO-related candidate genes in osteoporosis. **(A)** Volcano plot of DEGs in the OP and CON groups. **(B)** Overview heatmap of DEG expression in the GSE7429 circulating B-lymphocyte dataset. Individual heatmap rows are not assigned gene identities because the original row identifiers and clustering order are unavailable for verification. For labeled expression profiles of selected candidates (MTOR, AKT1, CCNE1, CES2, and ESR1), see Figure 3G; these profiles do not identify particular rows in panel B. **(C)** Overlap of OP-associated DEGs and predicted NEO targets, yielding 19 candidate genes. **(D)** GO enrichment of the candidate genes, including regulation of TORC2 signaling and negative regulation of autophagy. These annotations do not constitute measurements of mTORC1 or mTORC2 activity. **(E)** PPI network of the candidate genes after removal of isolated nodes. OP, osteoporosis; CON, high-BMD comparison group; DEGs, differentially expressed genes; GO, Gene Ontology; PPI, protein–protein interaction.

**FIGURE 3:**
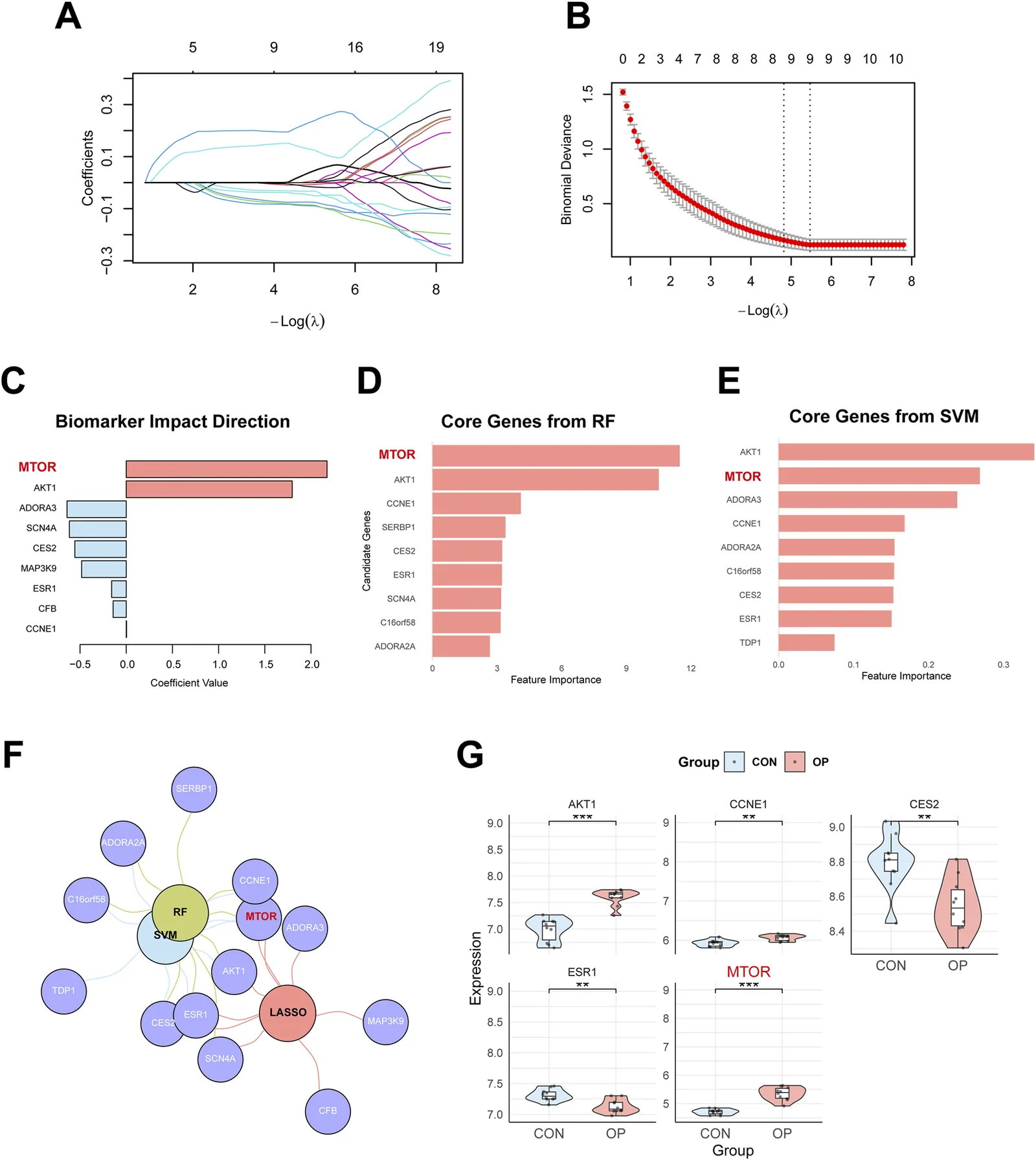
Machine-learning-based prioritization of MTOR as a candidate target in osteoporosis. **(A,B)** LASSO coefficient profiles and 10-fold cross-validation for selection of the penalty parameter λ. **(C)** Coefficients of candidate genes retained by LASSO regression. **(D,E)** Feature-importance rankings generated by RF and SVM analyses. **(F)** Network visualization of candidate-gene overlap among LASSO, RF, and SVM analyses, showing MTOR as the only candidate shared by all three methods. **(G)** Expression distributions of selected candidate genes, including MTOR, AKT1, CCNE1, CES2, and ESR1, in the CON and OP groups of the GSE7429 dataset.

After duplicate removal, the target-prediction platforms yielded 296 predicted NEO targets. Intersection with OP-associated DEGs identified 19 candidate genes (Figure 2C). GO enrichment analysis of these genes included regulation of TORC2 signaling, negative regulation of macroautophagy, negative regulation of autophagy, and cellular response to oxidative stress (Figure 2D). The TORC2-related annotation indicates enrichment of genes associated with that biological process; it is not an experimental measurement of mTORC2 activity or evidence of selective modulation of either mTOR complex by NEO. The autophagy-related terms supported subsequent pathway investigation.

The PPI network included AKT1, MTOR, ESR1, and IGF1R among the connected candidate proteins (Figure 2E). These network associations, together with the enrichment results, provided a rationale for further investigation of mTOR-associated signaling, without establishing direct drug–target binding.

From the analysis presented in the above paragraphs, we predicted that NEO might influence the osteogenic differentiation of BMSCs via the mechanism of mTOR-mediated autophagy pathway. Further experimental validation was thus conducted according to this prediction.

### 3.3 NEO alleviates DEX-induced inhibition of osteogenic differentiation in BMSCs by activating autophagy

Based on the bioinformatics findings, we tested whether NEO-associated restoration of osteogenic differentiation was accompanied by changes in autophagy.

Western blot analysis showed that DEX decreased the LC3B-II/LC3B-I ratio and increased p62 expression in BMSCs. High-dose NEO (NEO-H) partially reversed these changes (Figures 4A–C), consistent with restoration of autophagic activity.

**FIGURE 4:**
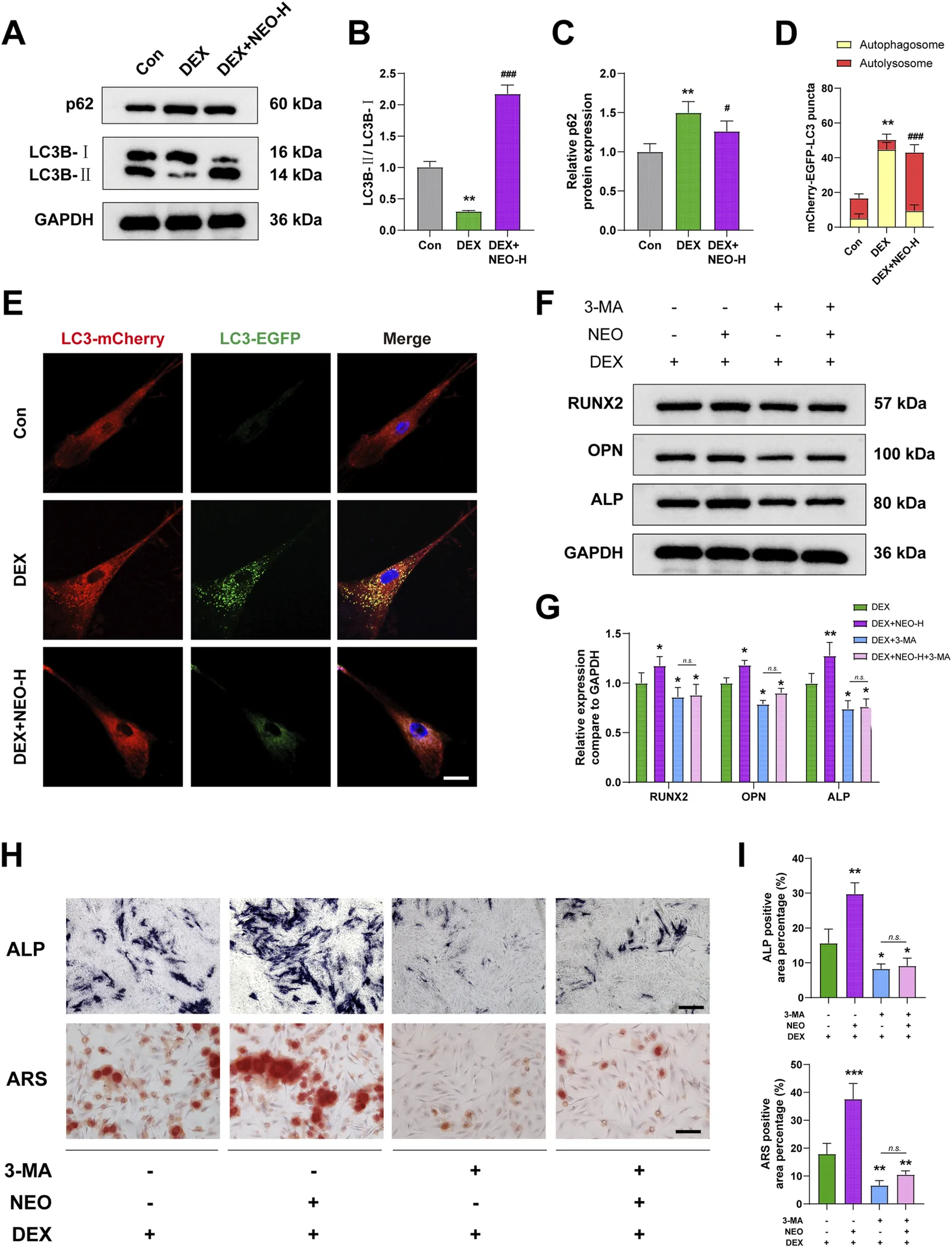
Neoeriocitrin ameliorates DEX-suppressed osteogenic differentiation in BMSCs via enhancing autophagy. **(A–C)** Western blot analysis and quantification of LC3B and p62 protein expression. **(D,E)** Representative images and quantitative analysis of autophagic flux using the mCherry-EGFP-LC3 reporter. Yellow: autophagosomes; red: autolysosomes. Autophagic flux was quantified by counting red-only (autolysosome) puncta. Scale bar: 20 μm. **(F,G)** Western blot of osteogenic markers (RUNX2, OPN, ALP) with/without 3-MA treatment. **(H,I)** Representative images and quantification of ALP and ARS staining in BMSCs under the indicated treatments. Scale bar: 100 μm. Values are presented as mean ± SD (n = 3 independent experiments). Statistical analysis was performed using one-way ANOVA followed by *post hoc* tests. **P < 0.01 vs. the Con group and #P < 0.05, ###P < 0.001 vs. the DEX group in panels B–D. *P < 0.05, **P < 0.01, ***P < 0.001 vs. the DEX group in panels G and I. n.s., not significant.

The mCherry-EGFP-LC3 reporter was used to assess autophagic flux. EGFP fluorescence is quenched in acidic autolysosomes, whereas mCherry fluorescence persists; yellow puncta therefore represent autophagosomes, and red-only puncta represent autolysosomes. DEX reduced the number of red-only puncta, whereas NEO increased it (Figures 4D,E), consistent with improved autophagic flux.

These findings suggest that NEO attenuates DEX-induced suppression of osteogenic differentiation in BMSCs by enhancing autophagic flux. This further supports the bioinformatics-based hypothesis that NEO acts through regulation of the autophagy pathway.

### 3.4 Complementary machine-learning analyses prioritized MTOR as a candidate target

To prioritize candidate genes for subsequent mechanistic investigation, the 19 genes obtained from the intersection analysis were evaluated using LASSO regression, RF, and SVM. These algorithms were applied as complementary feature-selection and feature-ranking approaches rather than as diagnostic classification models.

The LASSO coefficient profile and cross-validation curve are shown in Figures 3A,B. At the selected λ value, a subset of candidate genes was retained, including MTOR (Figure 3C). RF-based feature-importance analysis ranked MTOR first, followed by CCNE1, CES2, and ESR1 (Figure 3D). SVM analysis also placed MTOR among the top-ranked candidate genes (Figure 3E).

Comparison of the genes prioritized by the three methods showed that MTOR was the only candidate shared by the LASSO, RF, and SVM results (Figure 3F). The convergence of these complementary approaches supported the prioritization of MTOR for downstream mechanistic investigation. Because the analysis was conducted on a limited set of preselected candidate genes, these results were interpreted as supportive prioritization rather than independent machine-learning-based target discovery. However, this analysis was not intended to establish diagnostic performance or direct target engagement.

Expression comparison using the GSE7429 dataset showed that MTOR transcript abundance differed between the OP and CON groups. Other candidate genes, including AKT1, CCNE1, CES2, and ESR1, also showed differences between the two groups (Figure 3G). Because the same dataset was used for candidate-gene selection and expression comparison, these findings were considered descriptive support rather than independent validation.

Taken together with the pathway-enrichment and PPI-network results, the consistent prioritization of MTOR across the three algorithms provided a rationale for selecting MTOR for subsequent molecular docking, CETSA, pathway-activity analysis, and functional rescue experiments.

### 3.5 NEO regulates osteogenic differentiation through mTORC1-associated S6K1 signaling modulation

After mTOR was prioritized as a candidate target of NEO, molecular docking and cellular thermal stability analyses were performed to further investigate the potential association between NEO and mTOR and its regulatory effects on mTOR signaling.

Molecular docking using the 3FAP structural complex predicted a favorable binding pose of NEO, with a predicted docking energy of −10.0 kcal/mol. The predicted binding pose was located at the FKBP12–mTOR FRB interface, involving residues from both FKBP12 (chain A) and the mTOR FRB domain (chain B). On the mTOR side, the predicted interaction region included Thr187, Trp190, and Asp191, corresponding to Thr2098, Trp2101, and Asp2102 in the canonical human mTOR sequence, respectively. These residue assignments describe the reported pose; they do not define experimentally confirmed contacts or establish hydrogen-bond identities. These findings suggest a potential interaction of NEO with the FKBP12–mTOR FRB structural interface (Figure 5A).

**FIGURE 5:**
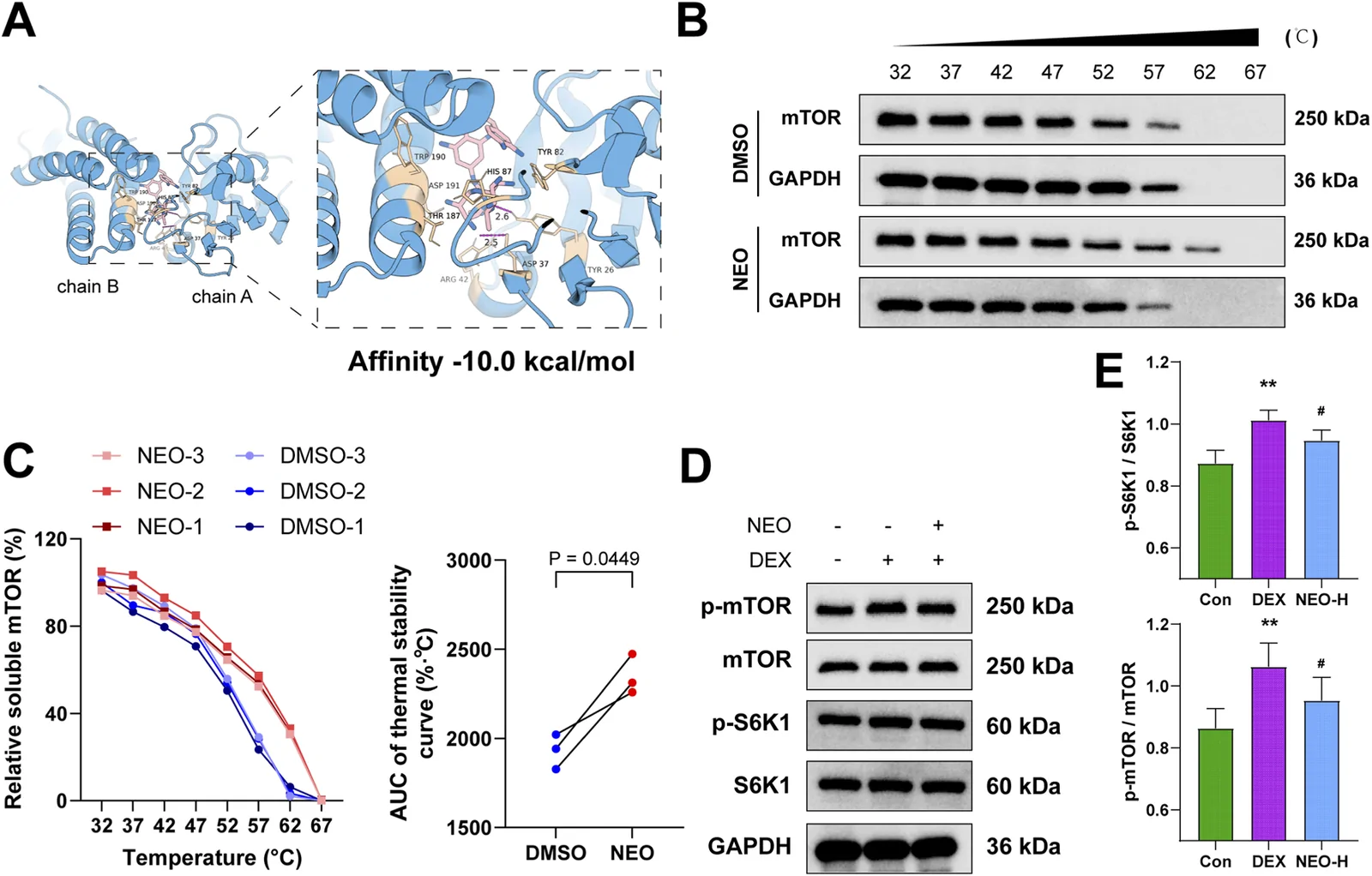
Neoeriocitrin alters mTOR thermal stability and suppresses DEX-induced mTORC1-associated S6K1 signaling in BMSCs. **(A)** Molecular docking simulation showing the predicted binding pose of NEO at the FKBP12–mTOR FRB interface (PDB ID: 3FAP). Chain A represents FKBP12 and chain B represents the mTOR FRB domain. The right-hand view is an enlargement of the reported three-dimensional pose, not a validated two-dimensional interaction map. Reported chain B residues Thr187, Trp190, and Asp191 correspond to canonical mTOR Thr2098, Trp2101, and Asp2102, respectively. These labels specify residue identities; interaction types and atom-specific contacts are not assigned without the original docked coordinates. **(B)** Representative CETSA Western blots showing soluble mTOR levels in DMSO- and NEO-treated BMSCs at 32 °C–67 °C. **(C)** Individual mTOR thermal stability curves from three independent experiments and paired AUC analysis, with mTOR signals normalized within each treatment group. **(D,E)** Western blot analysis of mTOR, p-mTOR (Ser2448), S6K1, and p-S6K1 (Thr389) protein expression. S6K1 Thr389 phosphorylation is an mTORC1-associated readout; mTOR Ser2448 phosphorylation alone does not distinguish mTOR complexes. Data are presented as mean ± SD (n = 3 independent experiments). **P < 0.01 vs. the Con group; #P < 0.05 vs. the DEX group.

CETSA was subsequently performed to evaluate whether NEO altered the intracellular thermal stability of mTOR. Soluble mTOR levels progressively decreased with increasing temperature in both groups; however, NEO-treated cells retained higher levels of soluble mTOR over the examined temperature range than DMSO-treated cells (Figures 5B,C). Analysis of three independent experiments showed that the area under the mTOR thermal stability curve was significantly increased following NEO treatment compared with DMSO treatment (P = 0.0449). These results indicate that NEO increases the cellular thermal stability of mTOR and are consistent with an intracellular mTOR-associated effect. They do not independently establish direct binding or identify which mTOR complex is affected.

Western blot analysis showed that DEX increased the p-mTOR (Ser2448)/mTOR and p-S6K1 (Thr389)/S6K1 ratios relative to the control group (both P < 0.01). NEO-H reduced both ratios relative to the DEX group (both P < 0.05; Figures 5D,E). The decrease in S6K1 Thr389 phosphorylation is consistent with reduced mTORC1-associated downstream signaling. Because mTORC2-specific readouts were not measured, these results cannot establish that NEO selectively affects mTORC1 or excludes an effect on mTORC2.

Together, the docking prediction, CETSA findings, and phosphorylation changes support an mTOR-associated mechanism involving the mTORC1–S6K1 signaling arm, autophagy, and osteogenic differentiation in BMSCs. Complex-selective target engagement remains unresolved.

### 3.6 Overexpression of mTOR attenuates the promotive effects of NEO on both osteogenic differentiation and autophagy in BMSCs

To further examine the functional contribution of mTOR, BMSCs were transfected with oe-mTOR or the corresponding empty-vector control for rescue experiments.

First, the results of ALP and ARS staining showed that after treating the oe-NC group with NEO, the DEX-inhibited osteogenic differentiation of BMSCs was effectively rescued, which was demonstrated by increased ALP activity and calcium mineralization. In contrast, in cells with mTOR overexpression, the osteoprotective properties of NEO were greatly reduced, along with an observed decrease in ALP-stained areas and mineralized nodules. The results suggest that mTOR overexpression greatly reduces the stimulatory activity of NEO towards osteogenesis (Figures 6A–C).

**FIGURE 6:**
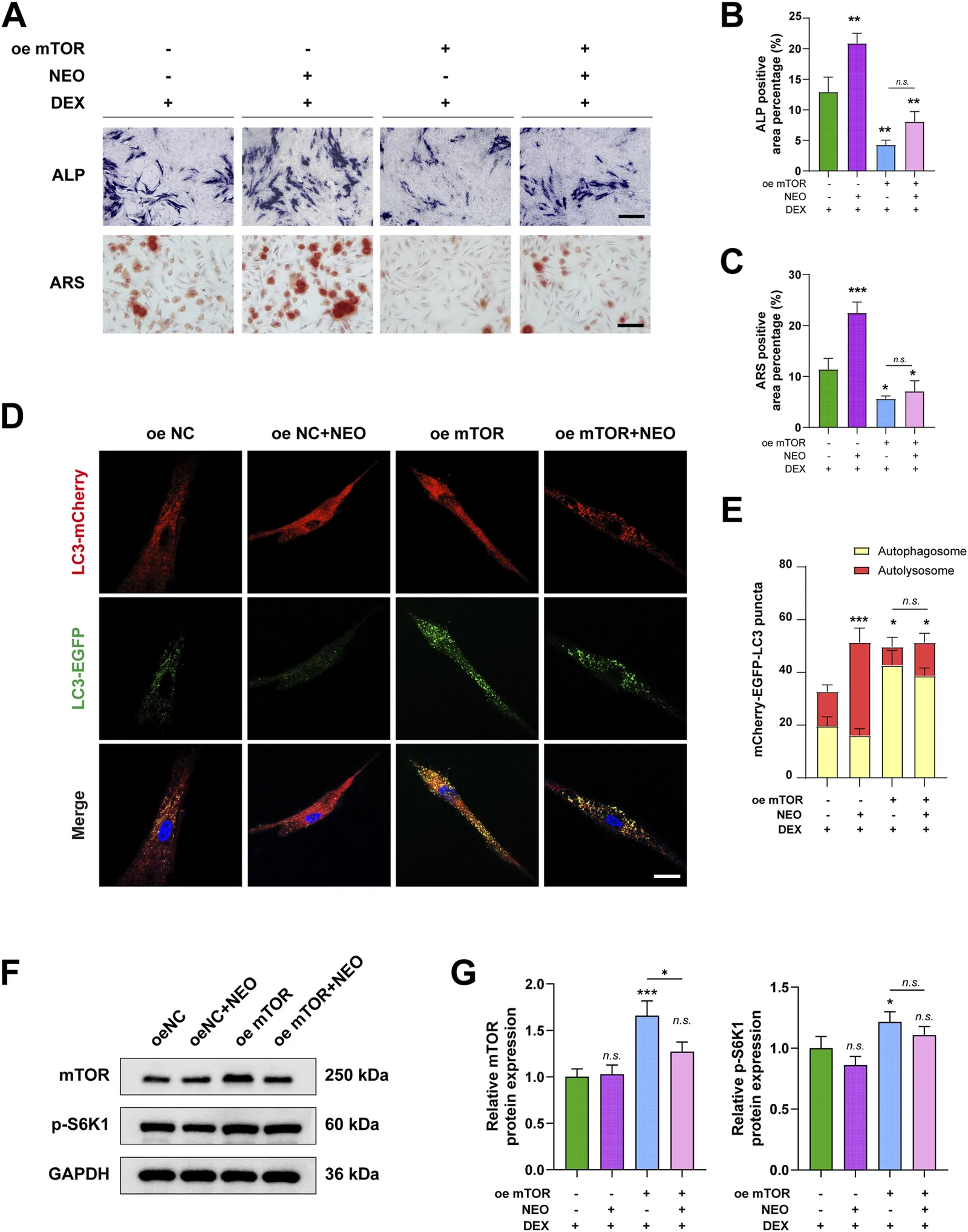
mTOR overexpression attenuates the pro-osteogenic and pro-autophagic effects of neoeriocitrin in BMSCs. **(A–C)** ALP/ARS staining and quantification in BMSCs. **(D,E)** Autophagic flux assay (mCherry-EGFP-LC3) and quantification in oe-NC/oe-mTOR BMSCs. **(F,G)** Western blot analysis of mTOR and p-S6K1 expression in oe-NC/oe-mTOR BMSCs. Data are presented as mean ± SD (n = 3 independent experiments). *P < 0.05, **P < 0.01, ***P < 0.001 vs. the DEX group. n.s., not significant.

Next, the dual reporter system of mCherry-EGFP-LC3 was used to examine the alterations in autophagic flux. It was found that in the oe-NC cells, treatment with NEO caused an increase in the number of red autolysosomes, indicating increased autophagic flux activity. Nevertheless, in mTOR overexpressed cells, despite treatment with NEO, the number of red dots was significantly reduced, and autophagic flux was significantly inhibited (Figures 6D,E). These results indicate that mTOR overexpression attenuated the pro-autophagic effect of NEO.

Western blot analysis showed that NEO treatment tended to reduce p-S6K1 levels in oe-NC cells. mTOR overexpression was associated with an increase in p-S6K1 levels and partially attenuated the NEO-associated reduction; however, most between-group comparisons did not reach statistical significance (Figures 6F,G). These findings indicate a trend toward attenuation of NEO-associated S6K1 suppression by mTOR overexpression rather than a robust rescue effect.

Overall, the functional rescue experiments further support an important functional role of mTOR signaling in mediating the effects of NEO on autophagy and osteogenic differentiation. The findings suggest that mTOR overexpression attenuates the regulatory effect of NEO on the mTOR/S6K1 signaling axis, although the changes in p-S6K1 were relatively modest.

## 4 Discussion

OP arises from an imbalance in bone remodeling between formation and resorption, and impaired osteogenic differentiation of BMSCs is a major contributor to reduced bone formation (Zhang K. et al., 2025). Glucocorticoid-induced osteoporosis is a type of secondary osteoporosis commonly observed in clinical settings. In this condition, DEX disrupts osteogenic signaling, increases oxidative stress, and impairs cellular homeostasis, thereby continuously weakening the osteogenic potential of BMSCs (Li et al., 2013; Zhou et al., 2014). Accordingly, a DEX-induced BMSC osteogenic inhibition model was established to investigate the osteoprotective effects of NEO. The results indicated that NEO significantly alleviated DEX-induced suppression of osteogenic differentiation, as evidenced by concurrent improvements in early osteogenic activity and late-stage mineralization capacity. This was accompanied by the restoration of osteogenesis-related protein expression, including RUNX2, OPN, and ALP. These findings not only support the osteogenic-promoting activity of NEO, but also indicate that its regulatory effects act at the level of osteoprogenitor cells. This provides more mechanistic evidence supporting the potential application of NEO in osteoporosis intervention.

Recently, growing attention has been focused on the involvement of autophagy in maintaining bone metabolic homeostasis (Levine and Kroemer, 2019). Moderate autophagy contributes to the maintenance of organelle turnover, energy metabolism balance, and a stable microenvironment for differentiation in BMSCs, thereby ensuring proper osteogenic differentiation. In contrast, impaired autophagy causes the accumulation of damaged organelles and metabolic waste, which ultimately compromises cellular differentiation capacity (Nollet et al., 2014). In the present study, DEX treatment was found to suppress autophagic flux in BMSCs, whereas NEO intervention effectively reversed this impairment. More importantly, inhibition of autophagy using 3-MA greatly attenuated the pro-osteogenic effects of NEO, supporting the involvement of autophagy in the osteogenic-promoting actions of NEO. Because 3-MA has effects beyond autophagy inhibition, these pharmacological findings support pathway involvement but do not establish an exclusive mechanism.

mTOR is a central regulator of cellular metabolism and autophagy (Chen and Long, 2018). mTORC1 controls autophagy initiation through substrates including ULK1, while S6K1 phosphorylation provides a downstream signaling readout (Saxton and Sabatini, 2017; Kim et al., 2011). The enrichment results included a TORC2-related biological process and autophagy-related terms, but enrichment alone does not identify the active mTOR complex. In contrast, the experimental pathway analysis focused on mTOR phosphorylation at Ser2448 and S6K1 phosphorylation at Thr389. Reduced S6K1 Thr389 phosphorylation after NEO treatment, together with improved autophagic flux, supports involvement of the mTORC1-associated signaling arm. This interpretation does not imply that S6K1 phosphorylation directly mediates all effects on autophagy. No AKT Ser473 assay or complex-specific perturbation was performed, so mTORC2 involvement and complex-selective effects of NEO remain unresolved.

Another noteworthy finding of this study is the potential association between NEO and mTOR signaling. Molecular docking predicted a favorable interaction of NEO at the FKBP12–mTOR FRB interface, while CETSA demonstrated increased cellular thermal stability of mTOR following NEO treatment. Quantification across three independent experiments further showed a significant increase in the AUC of the mTOR thermal stability curve. In parallel, NEO significantly suppressed DEX-induced increases in mTOR and S6K1 phosphorylation. Together with the functional rescue experiments using mTOR overexpression, these findings support an important role of mTOR signaling in mediating the effects of NEO on autophagy and osteogenic differentiation. Nevertheless, molecular docking and CETSA do not independently establish direct biochemical binding or direct inhibition of mTOR kinase activity, and further biophysical and enzymatic studies are required to characterize the precise molecular interaction.

This study extends previous work on NEO in osteogenic cell models by examining DEX-induced impairment of BMSC differentiation and integrating computational prioritization with docking, CETSA, signaling analysis, and functional rescue experiments. Several limitations constrain interpretation. First, no *in vivo* validation was performed. The cell-culture findings cannot establish improvements in bone mass, microarchitecture, mechanical strength, or fracture risk, and do not establish systemic safety. Further studies in glucocorticoid-induced and ovariectomy-associated bone-loss models should evaluate these outcomes alongside exposure, bioavailability, dosing, and toxicity. Second, the machine-learning analyses used a small predefined candidate set and a circulating B-lymphocyte dataset; they provide prioritization rather than independent target discovery or validation in skeletal cells. Third, the measured signaling arm is most consistent with mTORC1-associated S6K1 activity, but mTOR overexpression and CETSA do not distinguish mTORC1 from mTORC2. Complex-specific perturbations and additional readouts, including AKT Ser473 phosphorylation, are needed to resolve their respective contributions. Fourth, the unlabeled heatmap cannot support row-specific gene interpretation without its original identifiers and clustering order; the labeled candidate-gene profiles in Figure 3G provide a more transparent basis for discussing individual genes.

In addition, concentration-dependent CETSA was not performed in the present study; therefore, the CETSA findings should be interpreted as evidence of altered mTOR thermal stability rather than definitive evidence of direct biochemical binding.

Overall, NEO attenuated DEX-induced impairment of BMSC osteogenic differentiation *in vitro*, with accompanying changes in autophagic flux and mTORC1-associated S6K1 signaling. These findings provide a rationale for additional mechanistic and *in vivo* studies rather than evidence of an established osteoporosis treatment.

## 5 Conclusion

NEO alleviates DEX-induced inhibition of osteogenic differentiation in BMSCs in a concentration-dependent manner under the conditions examined. This effect is associated with reduced mTORC1-associated S6K1 signaling and restored autophagic flux. Docking and CETSA provide complementary evidence of a potential mTOR-associated effect but do not establish direct biochemical binding or complex-selective inhibition. *In vivo* efficacy, systemic safety, and the contribution of mTORC2 remain to be determined.

## Data Availability

The raw data supporting the conclusions of this article will be made available by the authors, without undue reservation.
